# Cost-Effective Control of Infectious Disease Outbreaks Accounting for Societal Reaction

**DOI:** 10.1371/journal.pone.0136059

**Published:** 2015-08-19

**Authors:** Shannon M. Fast, Marta C. González, Natasha Markuzon

**Affiliations:** 1 Information and Decision Systems Division, The Charles Stark Draper Laboratory, Cambridge, MA, United States of America; 2 Department of Civil and Environmental Engineering, Massachusetts Institute of Technology, Cambridge, MA, United States of America; University of Waterloo, CANADA

## Abstract

**Background:**

Studies of cost-effective disease prevention have typically focused on the tradeoff between the cost of disease transmission and the cost of applying control measures. We present a novel approach that also accounts for the cost of social disruptions resulting from the spread of disease. These disruptions, which we call social response, can include heightened anxiety, strain on healthcare infrastructure, economic losses, or violence.

**Methodology:**

The spread of disease and social response are simulated under several different intervention strategies. The modeled social response depends upon the perceived risk of the disease, the extent of disease spread, and the media involvement. Using Monte Carlo simulation, we estimate the total number of infections and total social response for each strategy. We then identify the strategy that minimizes the expected total cost of the disease, which includes the cost of the disease itself, the cost of control measures, and the cost of social response.

**Conclusions:**

The model-based simulations suggest that the least-cost disease control strategy depends upon the perceived risk of the disease, as well as media intervention. The most cost-effective solution for diseases with low perceived risk was to implement moderate control measures. For diseases with higher perceived severity, such as SARS or Ebola, the most cost-effective strategy shifted toward intervening earlier in the outbreak, with greater resources. When intervention elicited increased media involvement, it remained important to control high severity diseases quickly. For moderate severity diseases, however, it became most cost-effective to implement no intervention and allow the disease to run its course. Our simulation results imply that, when diseases are perceived as severe, the costs of social response have a significant influence on selecting the most cost-effective strategy.

## Introduction

Despite progress in the fight against infectious diseases, they remain a persistent threat to global health, resulting in approximately 15 million deaths annually [1]. Moreover, as illustrated by the recent H1N1 pandemic, pathogens can easily and quickly spread from country to country, infecting millions. Both researchers and policy makers are therefore interested in finding cost-effective solutions to reduce the burden of infectious disease transmission.

Modeling studies have investigated the cost-effectiveness of a number of intervention strategies, including vaccination, anti-viral use, and social distancing [2–5]. These studies attempt to quantify the cost to society imposed by the spread of disease (such as death, disability, and lost productivity resulting from illness), and by the intervention itself (such as vaccines, anti-virals, and lost productivity resulting from social distancing). They then determine which strategy minimizes the overall cost of the outbreak. These studies have proven useful for comparing the efficacy of different types of interventions. Nevertheless, an essential cost of disease transmission has not yet been considered in modeling efforts—the cost of social response.

Social responses are societal reactions to disease outbreaks. They range from anxiety to riots, violence, or flight [6–10]. Economic effects are also frequently observed, including the collapse of the tourism industry [11], an important source of income for many countries. The economic impact of social responses can sometimes be substantial, far outpacing the costs of the disease itself. For example, the World Bank has estimated that fear-driven changes in behavior, especially reductions in workplace productivity, travel, and consumer spending, are responsible for the bulk of the economic impact of the Ebola epidemic in West Africa [12]. Similar observations have been made about the SARS epidemic lasting from 2002 to 2004 [11]. With only 8096 cases reported worldwide [13], the direct costs of treatment were small. Nevertheless, substantial economic losses were incurred due to reductions in travel and consumer spending, as well as reduced confidence in the markets.

Far from the rational actors presented in many mathematical models, humans tend to inflate the actual risk from novel or severe pathogens, which are perceived as unpredictable and uncontrollable [14]. More mundane diseases, while frequently still deadly, typically elicit little response. We have developed a network-based model anticipating social response to disease outbreaks [15]. Social interactions are the main driver of disease transmission, while social response spreads through both social and media influence. The model has been shown to replicate population-level social responses on historical outbreaks. In the current work, we use this model to conduct a cost-effectiveness analysis in which we explicitly account for the cost of social response. We show how the optimal level and timing of intervention are affected. We also explore how the intervention strategy changes if intervention elicits increased media attention, leading to a surge in social response.

## Methods

We designed a set of simulations to explore how the least-cost intervention strategy is affected by considering the cost of social response. We assumed that the total cost of an outbreak is the sum of the cost of the disease itself, the cost of the intervention, and the cost of social response. Our formulation differs from previous cost-effectiveness analyses, because we assumed that the spread of disease has a cost beyond the people infected, namely, the social response.

### Model of Disease and Social Response Transmission

The simulations were conducted using ALARM (AnaLytic Anxiety Response Model), a stochastic model of the joint transmission of disease and social response. ALARM has been calibrated to data from historical disease outbreaks with and without social response, achieving a good fit to population-level disease transmission and social response [15]. The ALARM disease model is a susceptible-infected-recovered model, implemented on a network, with nodes representing individuals and edges representing social connections. Individuals become infected via contact with infected neighbors on the network, with per-contact probability *p*. Infected individuals recover and become immune to reinfection *T*_*R*_ days after infection. The size of the susceptible population, structure of the disease network and the relative values of *p* and *T*_*R*_ determine the expected size of the outbreak.

**Fig 1:**
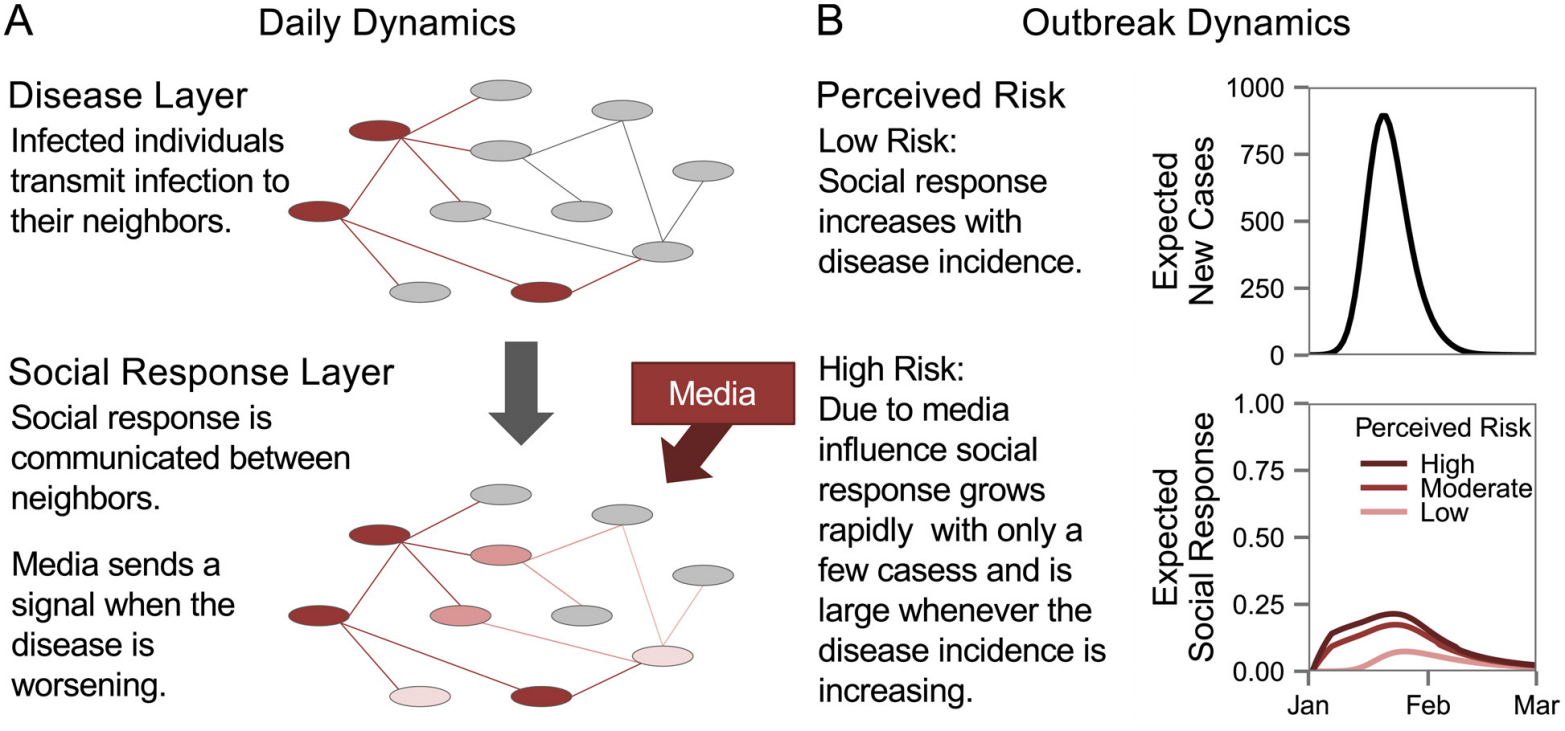
Overview of model dynamics. (A) Each day, infection spreads on the disease network, which sends a social response signal to the social response network. Social response is then transmitted on the social response network via social connections and a media signal. (B) Social response is primarily affected by the perceived risk of the disease. Outbreaks with the same number of cases produce different social responses depending upon whether the disease in question is perceived as low risk (such as seasonal influenza) or high risk (such as SARS).

The social response portion of the ALARM model connects the disease spread with the societal reaction. An individual *i*’s social response at each day *t*, *Y*_*i*_(*t*), is represented as a numeric value between 0 and 1, with 0 indicating no anxiety and 1 indicating extreme anxiety or behavioral manifestations of concern about disease, such as panic buying or participating in a protest. Social response is transmitted locally, via social connections, and globally, via a signal from the media. Infected individuals broadcast social response to their neighbors, who then communicate with their own neighbors spreading social response through the network. The media affects the social response independently of the magnitude of the disease spread. A key parameter of the model, *κ*, reflects the perceived risk of the disease. Higher values of *κ* indicate greater perceived risk, and, consequently, greater risk for social response if the disease spreads. Many factors affect the perceived risk of a disease, including its novelty and clinical severity, as well as the availability of counter-measures [7]. The complete methodology of ALARM is described in previous work [15] and in S1 File. A graphical summary of the model dynamics is provided in Fig 1.

### Approach to Determining the Most Cost-effective Level and Timing of Intervention

We define the cost of the outbreak as the sum of the costs of the disease spread, the intervention, and the social response. The costs of the disease spread are costs directly associated with caring for the infected, as well as the costs associated with mortality and lost workplace productivity. The costs of intervention are costs associated with vaccination, social distancing, and media campaigns intended to encourage preventive practices. Finally, the costs of social response include lost productivity, stress on the healthcare delivery system, and damage to property resulting from the population reaction to the outbreak. We define the expected cost of the disease, given the parameters, as the sum of the expected costs of disease spread, intervention, and social response:

$$\begin{array}{cc} \text{Expected}\;\text{Outbreak}\;\text{Cost} & =\;\text{Expected}\;\text{Cost}\;\text{of}\;\text{Infection}\\ & +\;\text{Expected}\;\text{Cost}\;\text{of}\;\text{Intervention}\\ & +\;\text{Expected}\;\text{Cost}\;\text{of}\;\text{Social}\;\text{Response.} \end{array}$$ (1)

More formally, let *c*_*D*_ be the expected cost resulting from one infected person. The the cost of the disease is *c*_*D*_ *ϕ*_*D*_ where *ϕ*_*D*_ represents the total number of people infected by the disease. Let the total cost of intervention be *c*_*I*_ *ϕ*_*I*_, where *ϕ*_*I*_ accounts for the level and duration of intervention, and let *c*_*S*_ *ϕ*_*S*_ be the cost of social response, where *ϕ*_*S*_ accounts for the social responses of the individuals in the community. We minimize the expected cost of the outbreak subject to three parameters: the timing of the intervention (*τ*), the duration of the intervention (*T*_*I*_), and the level of the intervention (*δ*). Let us denote the remaining parameters as *ψ*. We then define the expected cost of the outbreak as follows:

$$\begin{array}{cc} E(C|\psi ,T_{I},\tau & ,\delta )=\\ & c_{D}E(\phi_{D}|\psi ,T_{I},\tau ,\delta )+c_{I}E(\phi_{I}|\psi ,T_{I},\tau ,\delta )+c_{S}E(\phi_{S}|\psi ,T_{I},\tau ,\delta ). \end{array}$$ (2)

Using this formula, we can calculate the expected cost for any level (*δ*), duration (*T*_*I*_), and timing of intervention (*τ*). In the paper, we discuss the how *δ* and *τ* are chosen to minimize the cost for fixed *T*_*I*_. We present results showing the effects of *T*_*I*_ in S2 File.

### Implementation

#### Simulation Approach

We used a simulation approach to determine the most cost-effective social distancing strategy. Simulations were conducted on a scale-free network [16] with 30,000 nodes and a mean degree of 5 (household contacts [17] plus 2 to 3 additional contacts on average). We set the disease the per-contact probability of infection, *p*, such that a large proportion of the population, on average approximately 40%, would be infected. The time from infection to recovery, *T*_*R*_, was set to 6 days, reflecting the infectious period for influenza [18]. We explored six different scenarios, by varying the perceived risk of the disease and the media effect. In particular, for three values of the perceived risk of disease (low: *κ* = 0.50, moderate:*κ* = 0.75, and high: *κ* = 1.00), we found the most cost-effective social distancing strategy with and without the assumption that intervention leads to increased media attention. The following procedure was used to find the most cost-effective strategy for each scenario:

1. We ran 10,000 replications of the ALARM model for each parameter combination where *δ* ∈ {0.0, 0.2, …, 1.0}, *τ* ∈ {10, 1000, 5000}, and *T*_*I*_ ∈ {7, 21, 35}.
2. By averaging over the 10,000 replications, we obtained estimates of *E*(*ϕ*_*D*_ ∣ *ψ*, *T*_*I*_, *τ*, *δ*), *E*(*ϕ*_*I*_ ∣ *ψ*, *T*_*I*_, *τ*, *δ*), and *E*(*ϕ*_*S*_ ∣ *ψ*, *T*_*I*_, *τ*, *δ*).
3. We used Eq (2) to find the intervention strategy that minimized *E*(*C*∣*ψ*, *T*_*I*_, *τ*, *δ*) for each combination of *c*_*D*_, *c*_*I*_, and *c*_*S*_.

For our simulations, we defined *ϕ*_*D*_ as the total number of people infected in the outbreak. We defined *ϕ*_*I*_ with respect to the level of intervention (*δ*), size of the population (*n*), and the duration of the intervention (*T*_*I*_):

$$\begin{array}{c} \phi_{I}=\delta^{2}nT_{I}I\;(\phi_{D}\geq \tau ), \end{array}$$ (3)

where *I*(*ϕ*_*D*_ ≥ *τ*) is an indicator for the event in which the cumulative number of cases surpasses the intervention threshold, triggering the intervention. Finally, we defined *ϕ*_*S*_ as the sum of squared social responses. The social responses were squared to represent the fact that mild anxiety has almost no cost while severe social responses, such as rioting or seeking unnecessary medical treatment can be extremely costly. Specifically,

$$\begin{array}{c} \phi_{S}=\sum_{t=1}^{t_{L}}\sum_{i=1}^{n}Y_{i}{\left(t\right)}^{2}, \end{array}$$ (4)

where *t*_*L*_ is the last day of the outbreak and *Y*_*i*_(*t*) is individual *i*’s social response at day *t*. The parameters used in our simulations are listed in Tables 1 and 2.

**Table 1:** Disease transmission and social response parameters set at fixed values for all simulations.

**Parameter**	**Description**	**Value**	**Justification**
*p*	per-contact infection probability	0.07	40% of population infected, absent intervention
*T*_*R*_	duration of infective period	6 days	infectious period for influenza [18]
*κ*	disease risk index	0.50, 0.75, 1.00	explores the range of *κ* (0.5 ≤ *κ* ≤ 1.0)
*m*_*p*_	media penetration	0.1	estimated from historical outbreaks [21]
*q*	social interaction probability	0.5	assumed value; the model is insensitive to changes when *κ* > 0.5
*α*	response decay	0.95	estimated from historical outbreaks [15, 21]
$\bar{k}$	mean degree of scale-free network	5	assumed household contacts [17] and 2 to 3 additional contacts on average
*γ*	exponent of scale-free network	2.4	2 < *γ* < 3 for most real-world networks [16]

**Table 2:** Ranges of optimized disease transmission and social response parameters.

**Parameter**	**Description**	**Range**
*δ*	edge removal probability	0.0, 0.2, …, 1.0
*τ*	intervention threshold	10, 1000, 5000 cases
*T*_*I*_	duration of intervention	7, 21, 35 days

#### Social Response Resulting from Intervention

Several researchers have pointed to the phenomenon of social response resulting from the actions or communications of officials, rather than from the characteristics of the disease itself [19, 20]. By calling for an intervention, public officials draw additional media attention to the disease, making the public even more concerned and drawing attention away from other, possibly more pressing, public health concerns. We find the most cost-effective intervention strategy under this scenario, as well as under the scenario in which intervention has no cost in terms of social response and compare the results.

We modeled the surge in media attention resulting from the intervention by increasing both the magnitude of the media signal and the proportion of individuals in the community who receive the signal on the day on which the intervention was initiated. In the ALARM social response model, each individual typically receives a signal from the media of size *M*(*t*) with probability *m*_*p*_. On the day that the intervention begins, each individual receives a signal of size 5*M*(*t*) with probability tanh(10*m*_*p*_). Thus, on the day that the intervention begins, there is a higher probability of receiving a signal from the media and the magnitude of that signal is larger. On subsequent days, the media signal returns to normal. The parameters for the media surge were selected through analysis of data from the 2012–2013 influenza season in Boston, Massachusetts [21]. We ran the cost-effectiveness analysis with and without the assumption that intervention causes increased social response.

#### Social Distancing Intervention

A number of interventions can be considered for cost-effectiveness analysis. In the current implementation, we used social distancing as the intervention, simulating it through random edge removal in the network. In particular, after the cumulative number of cases surpassed the intervention threshold, *τ*, edges were randomly removed from the network, each with probability *δ*. After *T*_*I*_ days, the removed edges were restored to the network.

## Results

The simulations reveal that accounting for the cost of social response can dramatically alter the most cost-effective disease control strategy, when the disease has moderate to high perceived risk. When the disease has low perceived risk, the least-cost strategy is only marginally affected by the cost of social response. When intervention results in increased media attention, doing nothing frequently becomes the most cost-effective strategy. In the following sections, we present results for an intervention duration (*T*_*I*_) of 21 days and a cost coefficient of intervention (*c*_*I*_) of 0.05*c*_*D*_. In S2 File, we discuss the effects of varying the cost and duration of intervention. The cost coefficients of social response and intervention were defined relative to *c*_*D*_, the expected treatment cost of a single infected individual. Therefore, the expected costs of the simulated outbreaks are also defined relative to *c*_*D*_ (e.g. *E*(*C* ∣ *ψ*, *T*_*I*_, *τ*, *δ*) = 3.0*nc*_*D*_).

### Cost-Effective Intervention Assuming No Media Surge Resulting from Intervention

**Fig 2:**
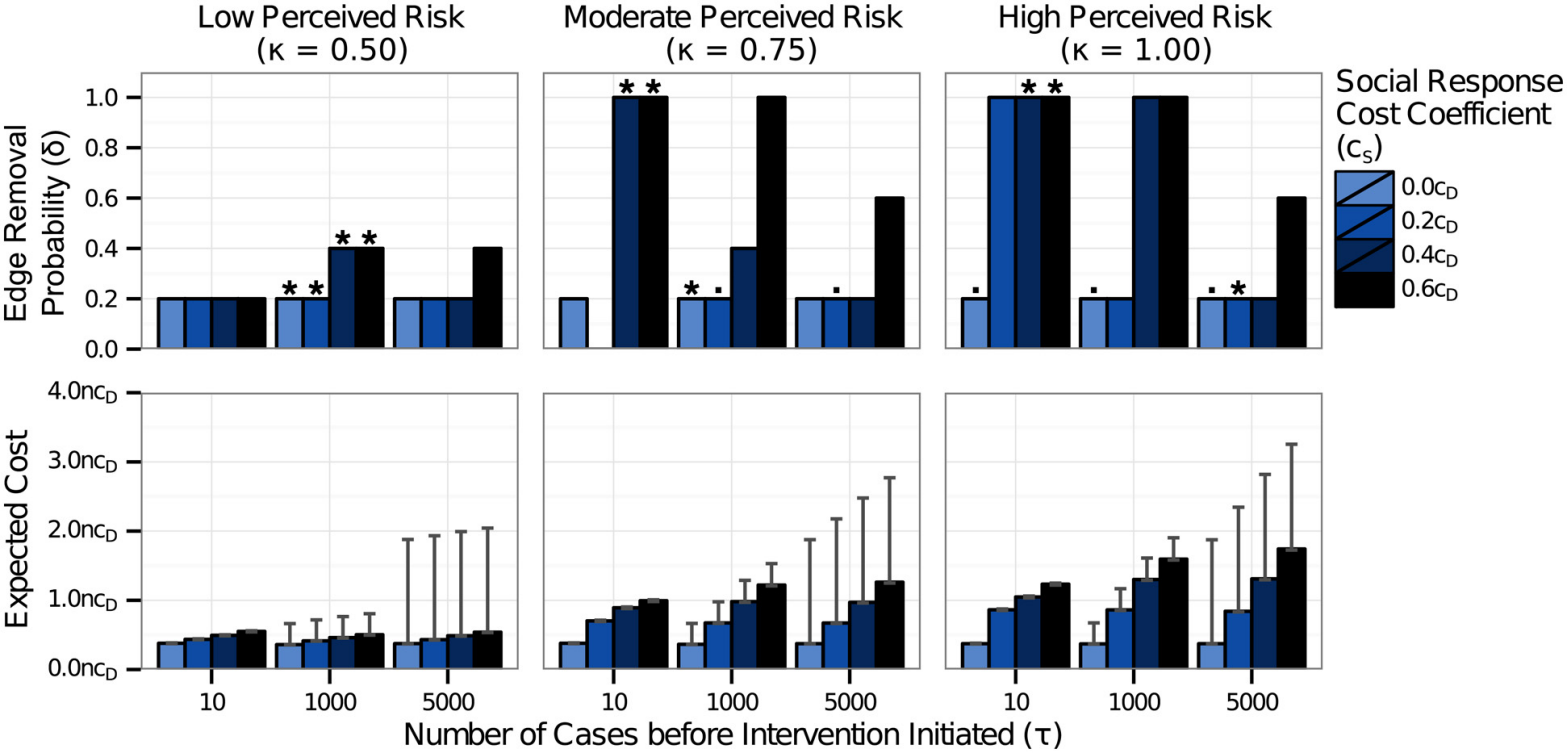
Most cost-effective intervention level and timing with no additional social response resulting from intervention. The least cost probability of edge removal and the associated expected cost are shown for three intervention thresholds (*τ* = 10 cases, 1000 cases, 5000 cases) and for four values of the cost coefficient of social response (*c*_*S*_). The cost coefficient of intervention (*c*_*I*_) was 0.05*c*_*D*_. The most cost-effective intervention is marked with a star (*) for each value of *c*_*S*_. In cases where the pairwise difference in expected cost between strategies was not statistically significant with a permutation test, all least-cost strategies are marked with dots (⋅). The error bars on the expected cost indicate the bootstrapped empirical 95% confidence interval. When no cost was assigned to the social response, the most cost-effective solution was to intervene at a low level as the outbreak approached its peak—here, after 1000 cases. As *c*_*S*_ increased, the most cost-effective strategy shifted toward intervening at higher levels, earlier in the outbreak. This effect was especially pronounced as the perceived risk of the disease (*κ*) increased.

In our simulations, the most cost-effective disease control strategy was strongly dependent upon the cost of social response and the perceived risk of the disease. Fig 2 shows the most cost-effective edge removal probability and timing, assuming that no additional social response results from the initiation of an intervention. When no cost was assigned to social response (*c*_*S*_ = 0.0*c*_*D*_), the cost was minimized by implementing a low level of social distancing (*δ* = 0.2) after the number of cases surpassed 1000 (Fig 2, top panels, light colored bars). This intervention had the effect of decreasing the disease incidence by 16%. While the social response was reduced by 18% for diseases with mild perceived risk, this intervention was not effective at decreasing the social response for diseases perceived as having moderate to high risk. In fact, the total social response increased by about 1% for such diseases. This increase occurred because the intervention was insufficient to quickly stop the outbreak. Even though the number of cases was reduced, the duration of the outbreak was slightly extended as a result of the intervention, leading to a longer period of media influence and more social response.

The cost of social response did not considerably affect the most cost-effective intervention strategy for diseases with low perceived risk (Fig 2, left panel). For diseases with moderate or high levels of perceived risk, the cost of social response played a meaningful role in determining the most cost-effective strategy. As the cost of social response increased, the most cost-effective strategy shifted toward intervening earlier in the outbreak, after only 10 cases, and at higher levels (Fig 2, middle and right panels). Our simulations reveal that, for diseases with moderate or high perceived risk, it can be worth the high costs of extreme intervention measures to stop transmission early in the outbreak and prevent the additional cases and social disruptions that would occur should the disease be allowed to spread.

**Fig 3:**
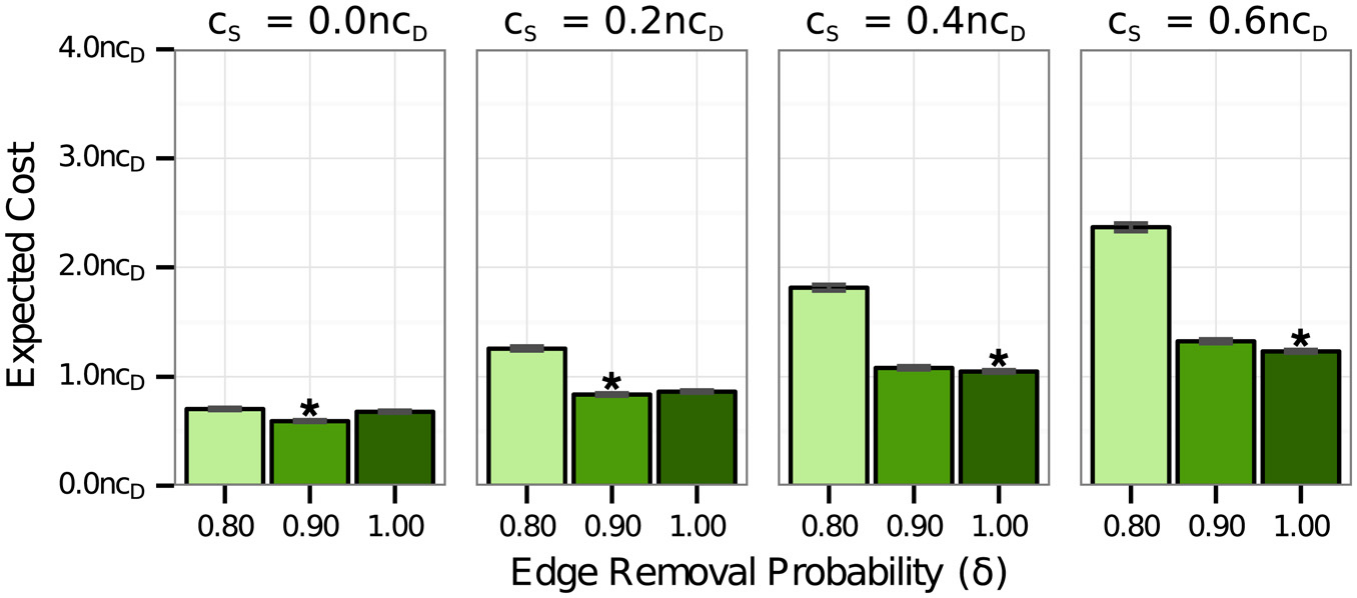
Cost of 80%, 90%, and 100% edge removal early in the outbreak. The expected costs are shown for a disease with high perceived risk (*κ* = 1.0) and interventions with 80%, 90%, and 100% edge removal all beginning after 10 cases of the disease. The cost coefficient for intervention (*c*_*I*_) was 0.05*c*_*D*_. The most cost-effective intervention for each value of *c*_*S*_ is marked with a star (*). In cases where the pairwise difference in expected cost between strategies was not statistically significant with a permutation test, all least-cost strategies are marked with dots (⋅). For *c*_*S*_ ≤ 0.2*c*_*D*_, 90% edge removal was significantly less costly than either 80% or 100% edge removal. For *c*_*S*_ ≥ 0.4*c*_*D*_, 100% edge removal was the most cost-effective intervention level. Nevertheless, 90% edge removal was still less costly than intervention later in the outbreak.

Our simulations show that 100% edge removal was frequently the least-cost solution when the perceived risk of disease was high. In general, social distancing interventions that are enacted early in an outbreak require a high level of social distancing to be effective. If the outbreak is not entirely stopped, the outbreak will be reseeded once the intervention ends, and the intervention will have limited effect [22]. Nevertheless, it is not necessarily true that there must be a complete shut down of activity in a population in order for the disease to be stopped early. In our original simulations, we examined values of edge removal spaced by 20% increments (0%, 20%, …, 100%). We conducted followup simulations in which intervention was implemented following 10 cases, and we compared the costs of 90% edge removal for diseases with high perceived risk (*κ* = 1.0) with the costs of 80% and 100% edge removal. We found that for lower values of the cost of social response, 90% edge removal was significantly less costly than either 80% or 100% edge removal (Fig 3, left panels). At high values of the cost of social response, 100% edge removal was less costly than 90% edge removal (Fig 3, right panels), though 90% edge removal was less costly than any level of intervention later in the outbreak. These findings suggest that intervention early in the outbreak can be effective, even when the social distancing is not absolute. It is important, however, to actually stop the transmission of disease. With 80% edge removal, the disease spread was stopped before 5000 cases in 56% of simulations. With 90% and 100% edge removal, the disease spread was stopped before 5000 cases in 93% and 100% of simulations, respectively. For this study, we used random edge removal as the social distancing intervention. In the early stages of the outbreak, social distancing targeted in the vicinity of infected individuals is likely to be effective and may be a less costly alternative to population-wide social distancing.

### Cost-Effective Intervention Assuming Media Surge Resulting from Intervention

**Fig 4:**
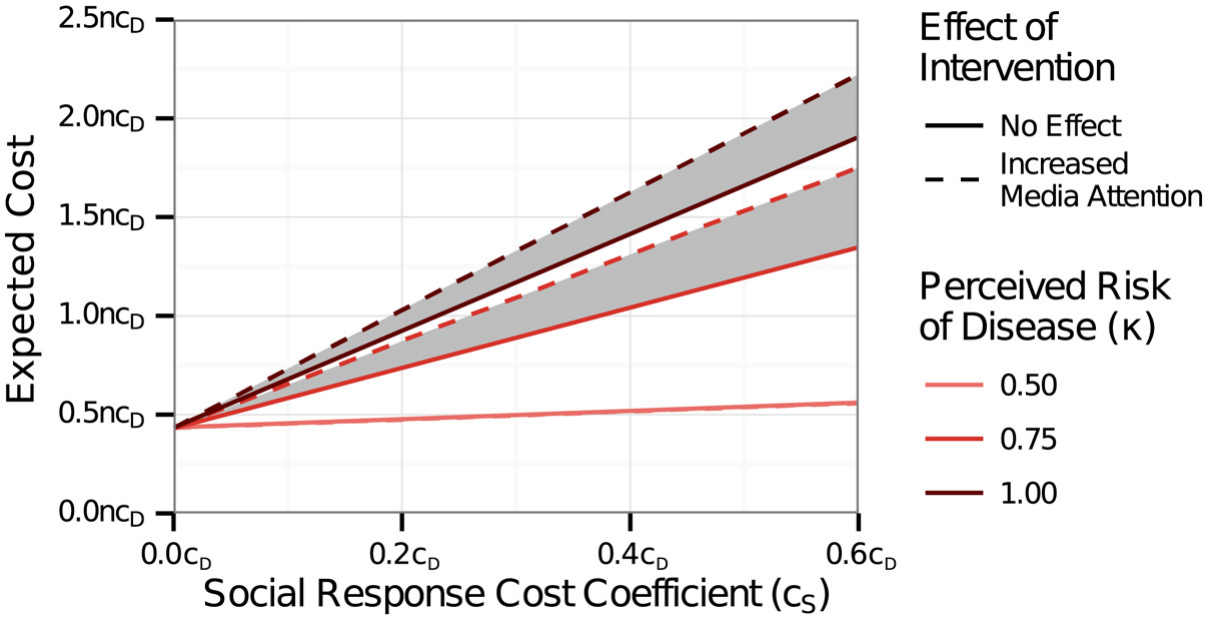
Cost of media attention resulting from the initiation of an intervention to control the disease. The expected cost is shown for an intervention initiated at 1000 cases with 40% edge removal. The cost coefficient for intervention (*c*_*I*_) was 0.05*c*_*D*_. The cost increased with the perceived risk of disease and with the social response cost coefficient (*c*_*S*_). When the perceived risk of the disease was low (*κ* = 0.50), the media attention resulting from the intervention did not increase the overall cost. When the perceived risk was higher, the cost was substantially increased by the media attention.

We ran the cost-effectiveness analysis again with the assumption that the decision to implement an intervention led to a temporary increase in media focus on the disease, and, as a result, increased social response. Fig 4 illustrates the effect of this assumption on the overall cost of the outbreak for an intervention initiated at 1000 cases with 40% edge removal. Under our assumptions about the effect of intervention on the the media penetration and signal, the increase in cost was proportionate to the social response cost coefficient for diseases with moderate or high perceived risk (*κ* ∈ {0.75, 1.00}). For diseases with low perceived risk, there was no additional social response.

**Fig 5:**
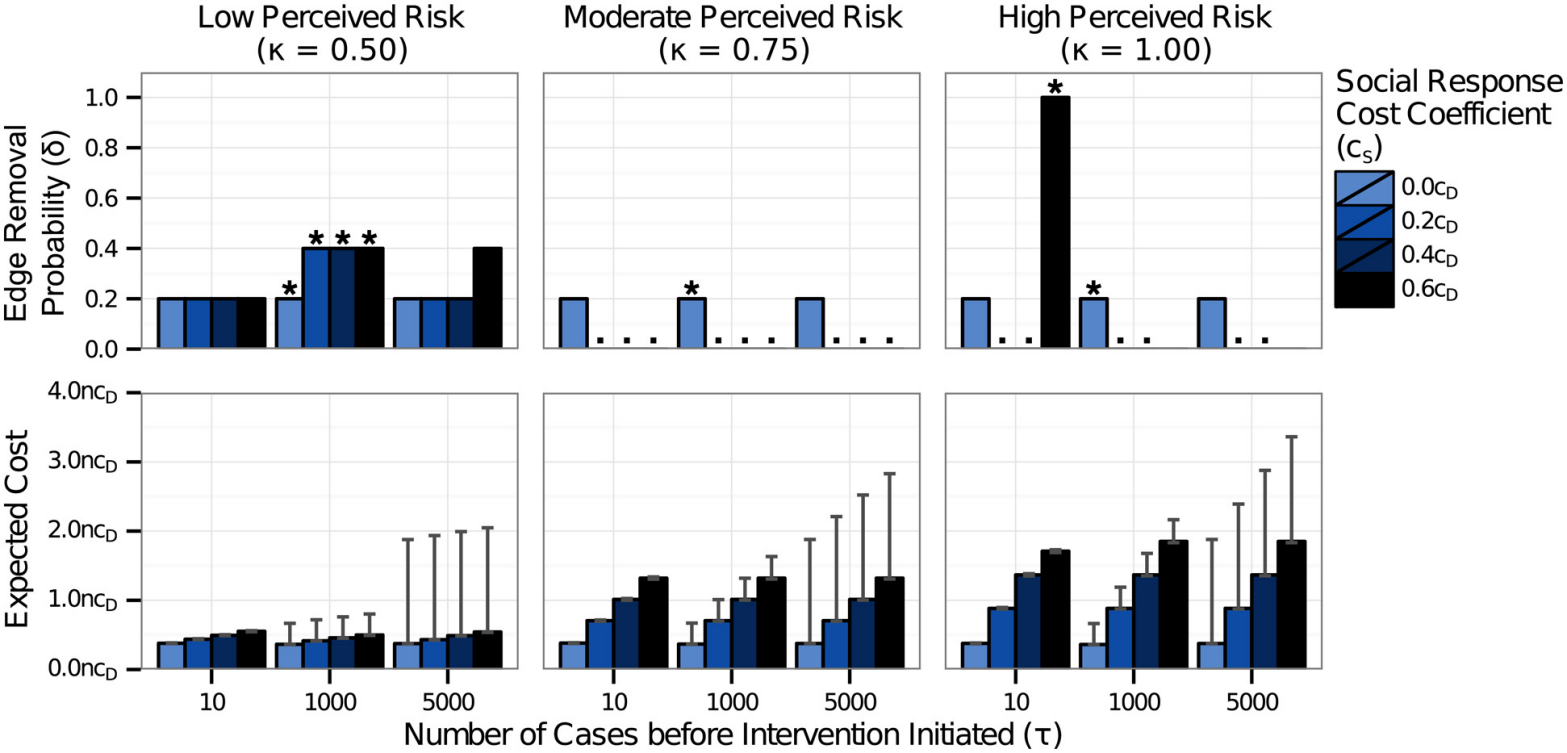
Most cost-effective intervention level and timing when intervention leads to a surge in media attention and social response. The least cost probability of edge removal and the associated expected cost are shown for three intervention thresholds (*τ* = 10 cases, 1000 cases, 5000 cases) and for four values of the cost coefficient of social response (*c*_*S*_). The cost coefficient for intervention (*c*_*I*_) was 0.05*c*_*D*_. The most cost-effective intervention is marked with a star (*) for each value of *c*_*S*_. In cases where the pairwise difference in expected cost between strategies was not statistically significant with a permutation test, all least-cost strategies are marked with dots (⋅). The error bars on the expected cost indicate the bootstrapped empirical 95% confidence interval. The most cost-effective intervention strategy was unchanged when the disease has low perceived risk (*κ* = 0.50). For diseases with moderate or high perceived risk, the least-cost strategy shifted to implementing no intervention, for most values of the cost coefficient of social response that we examined. For *c*_*S*_ = 0.6*c*_*D*_, it remained optimal to intervene following only 10 cases with 100% edge removal.

Fig 5 shows the most cost-effective level and timing of intervention, under the assumption that intervention temporarily increased media attention. When the cost of social response was considered (*c*_*S*_ > 0.0*c*_*D*_) and the perceived risk of the disease was moderate or high (*κ* ∈ {0.75, 1.0}), the most cost-effective solution was frequently to implement no intervention (Fig 5, middle and right panels). In these cases, the increased social response resulting from the intervention was not justified by the reduction in cases. For diseases with high perceived risk (*κ* = 1.0), the most cost-effective solution remained to intervene at a high level early in the outbreak, if the cost coefficient of social response was sufficiently high (Fig 5, right panel). In particular, when *c*_*S*_ = 0.06*c*_*D*_, we found that the least-cost solution for diseases with high perceived risk (*κ* = 1.0) was to implement 100% edge removal after 10 cases of disease. In contrast, the least-cost solution for diseases with moderate perceived risk (*κ* = 0.75) was to implement no intervention. The difference in strategies resulted from differences in the cost of allowing the disease to run its course. With no intervention, the expected cost for diseases with high perceived risk was 40% greater than that for diseases with moderate perceived risk. As a result, it was worth the cost of intervention for diseases with high perceived risk to stop the spread of disease quickly.

## Discussion

In our simulations, accounting for social response when evaluating how respond to an infectious disease outbreak, resulted in the use of increased disease control measures in the most cost-effective strategy. Moreover, the least-cost disease control strategy was highly dependent upon the perceived risk of the disease (*κ*). For diseases that are perceived to be mild, such as seasonal influenza, our analysis suggests that the most cost-effective solution is to implement moderate control measures. For diseases with moderate to high perceived severity, such as pandemic influenza or SARS, the most cost-effective strategy shifts toward intervening earlier in the outbreak and with more resources. Acute social response can be generated by only a few cases of these diseases, and controlling these diseases rapidly is of great importance in order to minimize the cost of the outbreak. The strategy of early intervention for severe diseases is common practice in public health already. Our study indicates that this strategy is likely to be effective, not only for controlling the disease, but also for preventing extreme societal reactions.

When intervention elicits increased media involvement, our simulations suggest that it remains important to control high severity diseases quickly. For moderate severity diseases, however, the most cost-effective situation shifts to implementing no intervention. The cost of the media frenzy surrounding the intervention can sometimes surpass the cost of a few more cases of the disease. Policy-makers should therefore carefully consider the possible costs of social response prior to implementing an intervention.

One difficulty in formulating a cost-effectiveness analysis including social response is that the cost coefficient of social response is unknown. With some research, it is possible to assign a dollar amount to the cost of treatment and lost productivity per infected person (*c*_*D*_). Similarly, it is possible to quantify the costs of an intervention (*c*_*I*_). Assigning a monetary value to social response is much more difficult. Choosing what cost to assign to the public’s fear is, at its heart, a value judgment. For this case study, we have selected to present the most cost-effective strategies for a range of possible cost coefficients, reflecting the fact that the coefficients chosen by a public health official, a politician facing a tough election, and an average person may not be the same. Despite the difficulties associated with quantifying the costs of social response, monetary estimates of the costs involved in social response will be needed in order to give more precise guidance to decision makers. Much of the current research into the economic effects of disease outbreaks does not separate the effects of social response from the effects of disease transmission and intervention. For example, many studies quantify losses to the tourism industry, but do not differentiate between losses resulting from fear of traveling (social response), and losses resulting from travel bans (intervention) [12, 23]. Similarly, some costs that appear to be costs of disease transmission, such as costs from visits to hospital emergency rooms, can partially result from social response. With high profile diseases, the worried well and worried ill place an increased burden on the health system in the form of unnecessary medical testing and care [24]. Further economic analyses will be needed in order to untangle the costs of social response from those of disease spread and intervention.

Our model simulations have shown that for diseases with moderate to high perceived risk, incorporating the cost of social response results in implementing a dramatically different intervention strategy than would be suggested by a cost-effectiveness analysis that does not consider the costs of social response. In order to make cost-effectiveness analysis useful in cases of these severe diseases, it will be important to appropriately quantify the cost of social response. Otherwise, cost-effectiveness analysis will not achieve its goal of controlling disease spread in the most efficient way possible. The spread of disease takes place in a dynamic social environment, and the costs of a disease outbreak are not limited to only treatment costs and disease control costs. Diseases can have much broader social impacts, including effects on the economy and social upheaval. This work represents a first step toward developing effective disease control measures, taking into account the indirect social costs of an outbreak. This type of model can aid analysts in assessing disease outbreaks and making informed decisions about application of control measures.

## Supporting Information

S1 File ALARM methodology. (PDF)

S2 File Results of varying the cost and duration of intervention. (PDF)
