# Supplementary material for: Cost-Effective Control of Infectious Disease Outbreaks Accounting for Societal Reaction
Source: PLoS One. 2015 Aug 19;10(8):e0136059. doi: 10.1371/journal.pone.0136059 (PMC4542207; doi:10.1371/journal.pone.0136059)
Supplement: S1 File — (PDF) [file pone.0136059.s001.pdf]

ALARM Methodology

Supporting Information for Cost-effective Control of Infectious Disease Outbreaks Accounting for Societal Reaction

Shannon M. Fast[1], Marta C. González[2], Natasha Markuzon[1,*]

1 Information and Decision Systems Division, The Charles Stark Draper Laboratory, Cambridge, MA, USA
2 Department of Civil and Environmental Engineering, Massachusetts Institute of Technology, Cambridge, MA, USA

*** nmarkuzon@draper.com**

Disease Transmission Dynamics

We implement an susceptible-infected-recovered (SIR) model [1], adapted for network-based modeling [2]. Each individual i's disease state at time t is represented by $X_i(t) \in \{S, I, R\}$, where S = susceptible, I = infected, and R = recovered. Infection is transmitted through pair-wise contact with infected neighbors on the disease network. At time t, an infected individual infects each of her susceptible neighbors, independently, with probability p. Thus, if $X_i(t) = I$, $X_j(t) = S$, and i and j are neighbors on the disease network, then:

$$X_j(t+1) = \begin{cases} I & \text{with probability } p \\ S & \text{with probability } 1-p. \end{cases} \tag{1}$$

Following infection, agents recover after T_R time periods. Therefore, if $X_i(t-1) = S$ and $X_i(t) = I$, then:

$$X_i(t) = \cdots = X_i\left(t + T_R - 1\right) = I, X_i\left(t + T_R\right) = R. \tag{2}$$

The disease will grow into an epidemic if the infection rate is sufficiently larger than the recovery rate [2].

Social Response Transmission Dynamics

The social response communication process at each day t has three steps. First, the social responses of individuals who were infected on day t are set to κ, via the function f. Thus,

$$f\left(Y_i(t-1)\right) = \begin{cases} \kappa & \text{if } i \text{ was infected at time } t \\ Y_i(t-1) & \text{otherwise.} \end{cases} \tag{3}$$

Then individuals communicate social response with their neighbors and receive a signal from the media. Following this step, individual i's social response is given by:

$$g\left(Y_i(t-1)\right) = \tanh\left(M_i(t) + \frac{1}{\sum_{j \in (i,j)} I_{ij} w_j} \sum_{j \in (i,j)} I_{ij} w_j f\left(Y_j(t-1)\right)\right) \tag{4}$$

where $M_i(t)$ is the media signal received by i, I_{ij} is an indicator for whether individuals i and j interact and w_j is a weight assigned to j's social response. The weights are used to give a bias toward paying attention to more concerned neighbors:

$$w_j(t) = \begin{cases} 10 & \text{if } Y_j(t) \geq 0.5 \\ 1 & \text{otherwise.} \end{cases} \tag{5}$$

The final step in the social response communication process is social response decay, reflecting the eventual decline in social response as the disease spread tapers off. Following this step, the social response of individual i is given by:

$$Y_i(t) = \alpha \times g\left(Y(t-1)\right), \tag{6}$$

where $\alpha \in [0, 1]$.

Media Influence

The global media signal at time t is given by $M(t)$. The probability of a given individual receiving a signal from the media on time t is m_p. Thus,

$$M_i(t) = \begin{cases} M(t) & \text{with probability } m_p \\ 0 & \text{with probability } 1 - m_p. \end{cases} \tag{7}$$

Let $N_I(t)$ be the number of people infected with the disease at time t. If the current number of infected individuals is greater than half the maximum observed previously in the outbreak, then the media sends a signal. Otherwise, no signal is sent. Thus,

$$M(t) = \begin{cases} 2\kappa - 1 & \text{if } N_I(t) > \frac{1}{2} \times \max\left\{N_I(0), \ldots, N_I(t-1)\right\} \\ 0 & \text{otherwise.} \end{cases} \tag{8}$$

In practice, this rule results in a media signal being sent when the disease is actively spreading in the population.

Implementation

The model is initiated with one infected individual. Then each time period, t, is simulated by simulating disease state transitions and then social response state transitions.

References

1. Kermack WO, McKendrick AG. Contribution to the mathematical theory of epidemics. Proc R Soc A. 1927; 115: 700-721. doi: 10.1098/rspa.1927.

2. Newman MEJ. Networks: An introduction. Oxford: Oxford University Press; 2010.
