# Supplementary material for: Cost-Effective Control of Infectious Disease Outbreaks Accounting for Societal Reaction
Source: PLoS One. 2015 Aug 19;10(8):e0136059. doi: 10.1371/journal.pone.0136059 (PMC4542207; doi:10.1371/journal.pone.0136059)
Supplement: S2 File — (PDF) [file pone.0136059.s002.pdf]

Results of Varying the Cost and Duration of Intervention

Supporting Information for Cost-effective Control of Infectious Disease Outbreaks Accounting for Societal Reaction

Shannon M. Fast[1], Marta C. González[2], Natasha Markuzon[1,*]

1 Information and Decision Systems Division, The Charles Stark Draper Laboratory, Cambridge, MA, USA
2 Department of Civil and Environmental Engineering, Massachusetts Institute of Technology, Cambridge, MA, USA

*** nmarkuzon@draper.com**

Effect of the Cost of Intervention

We examined three values of the cost coefficient of intervention (c_I): $0.03c_D$, $0.05c_D$, and $0.07c_D$. The effect of varying the cost coefficient of intervention was straightforward. When intervention was inexpensive, the least-cost solution involved intervening early in the outbreak and with high levels of edge removal. As the cost of intervention increased, intervention took place later with lower levels of edge removal or did not take place at all. Fig. S1 and Fig. S2 show the most cost-effective level and timing of intervention for $c_I = 0.03c_D$ and $c_I = 0.07c_D$ respectively. Results for $c_I = 0.05c_D$ are shown in the main text.

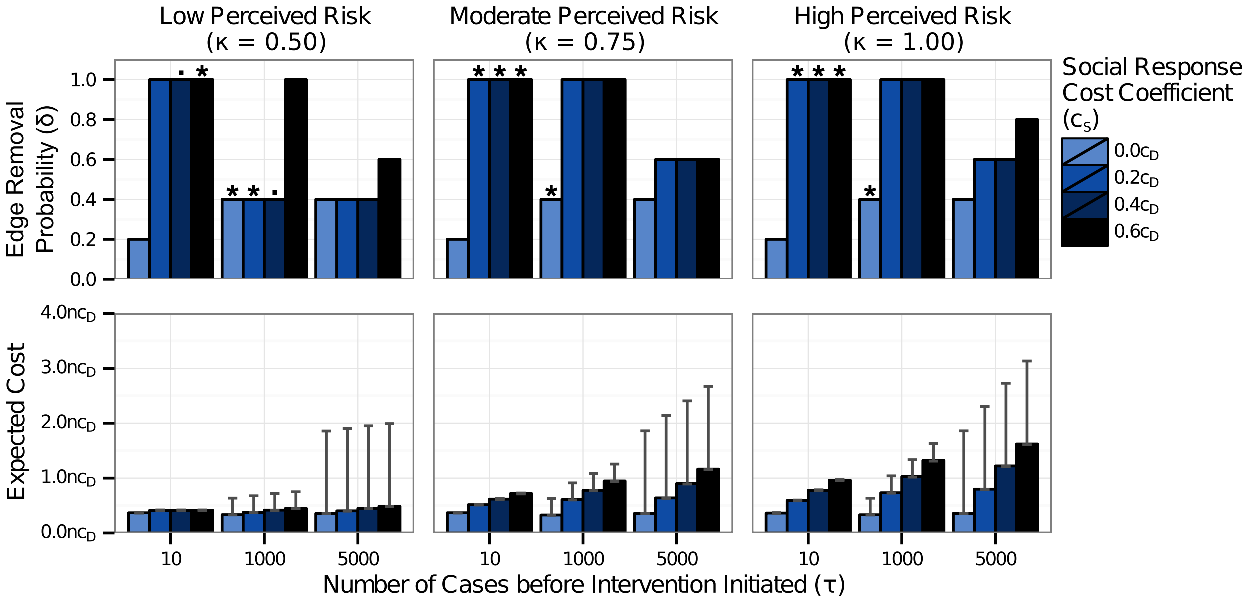

Figure S1. Most cost-effective intervention level and timing with no additional social response resulting from intervention for $c_I = 0.03c_D$. The least cost level of edge removal and the associated expected cost are shown for three intervention thresholds ($\tau = 10$ cases, 1000 cases, 5000 cases) and for four values of the cost coefficient of social response (c_S). The most cost-effective intervention is marked with a star (*) for each value of c_S. In cases where the pairwise difference in expected cost between strategies was not statistically significant with a permutation test, all least-cost strategies are marked with dots ($\cdot$). The error bars on the expected cost indicate the bootstrapped empirical 95% confidence interval.

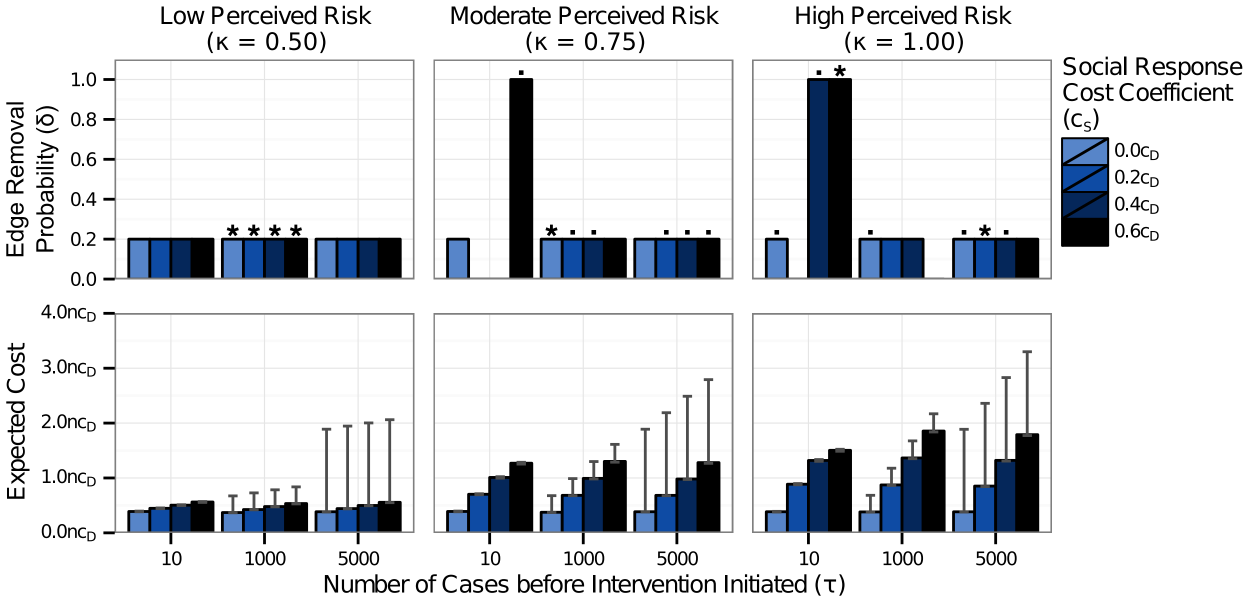

Figure S2. Most cost-effective intervention level and timing with no additional social response resulting from intervention for $c_I = 0.07c_D$. The least cost level of edge removal and the associated expected cost are shown for three intervention thresholds ($\tau = 10$ cases, 1000 cases, 5000 cases) and for four values of the cost coefficient of social response (c_S). The most cost-effective intervention is marked with a star (*) for each value of c_S. In cases where the pairwise difference in expected cost between strategies was not statistically significant with a permutation test, all least-cost strategies are marked with dots ($\cdot$). The error bars on the expected cost indicate the bootstrapped empirical 95% confidence interval.

Effect of the Duration of Intervention

We examined three values of the duration of intervention (T_I): 7, 21, and 35 days. Results for $T_I = 21$ are discussed in the main text. When the duration of intervention was short ($T_I = 7$), 100% edge removal was the least-cost solution for all levels of perceived risk. In our simulations, each infected individual remained infected for 6 days. Thus, a very high level of edge removal was necessary for the intervention to have an effect on the total number of cases. Lower levels of edge removal merely delayed the outbreak. This finding is consistent with research on the effects of school closure during outbreaks, which has shown that schools must be closed for an extended duration in order to be effective [1, 2].

When the duration of intervention was long ($T_I = 35$), the least-cost solution was to intervene early in the outbreak. When the perceived risk was low, 20% edge removal after 10 cases, was the least-cost solution. This early, low-level intervention was ineffective at stopping the disease early, with the disease spread continuing past 100 cases in 65% of simulations. Nevertheless, in the few simulations in which the intervention was effective, a large number of cases were averted with little cost. At higher levels of perceived risk, the gamble of a low-level intervention early in the outbreak was not worth the cost, because intervening early often delayed the onset of the outbreak, leading to more days of social response. As when the duration of intervention was 21 days, the least-cost solution when the perceived risk of disease was high ($\kappa = 1.0$) and the cost of social response was high ($c_S = 0.06c_D$) was to implement 100% edge removal after only 10 cases.

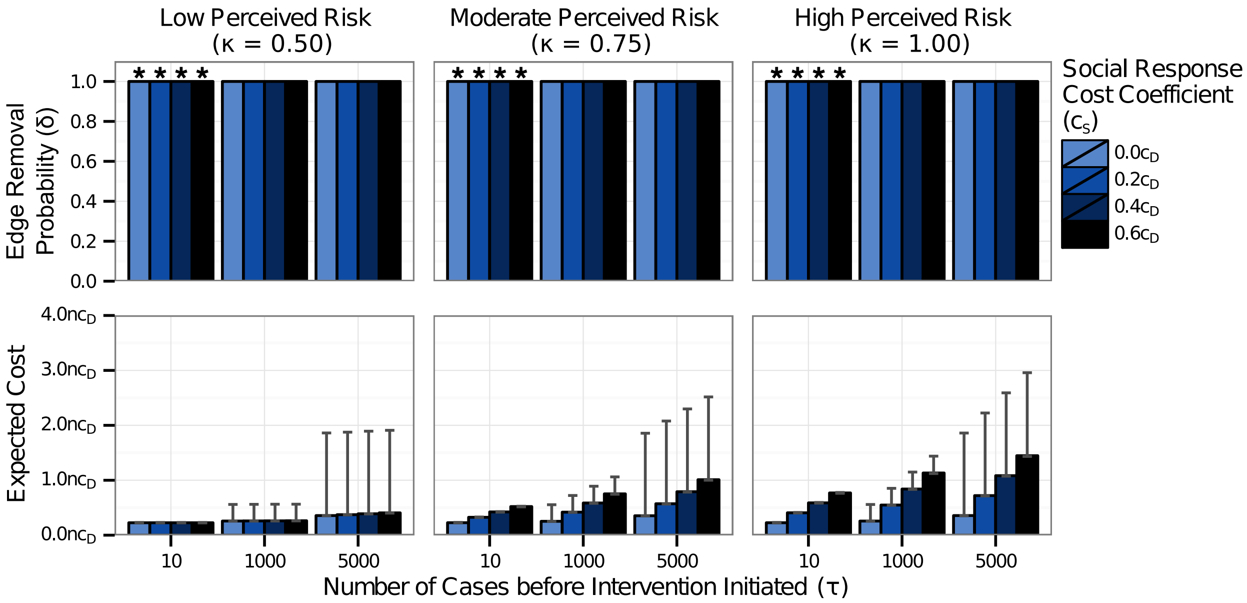

Figure S3. Most cost-effective intervention level and timing with no additional social response resulting from intervention for $T_I = 7$. The least cost level of edge removal and the associated expected cost are shown for three intervention thresholds ($\tau =$10 cases, 1000 cases, 5000 cases) and for four values of the cost coefficient of social response (c_S). The most cost-effective intervention is marked with a star (*) for each value of c_S. In cases where the pairwise difference in expected cost between strategies was not statistically significant with a permutation test, all least-cost strategies are marked with dots ($\cdot$). The error bars on the expected cost indicate the bootstrapped empirical 95% confidence interval.

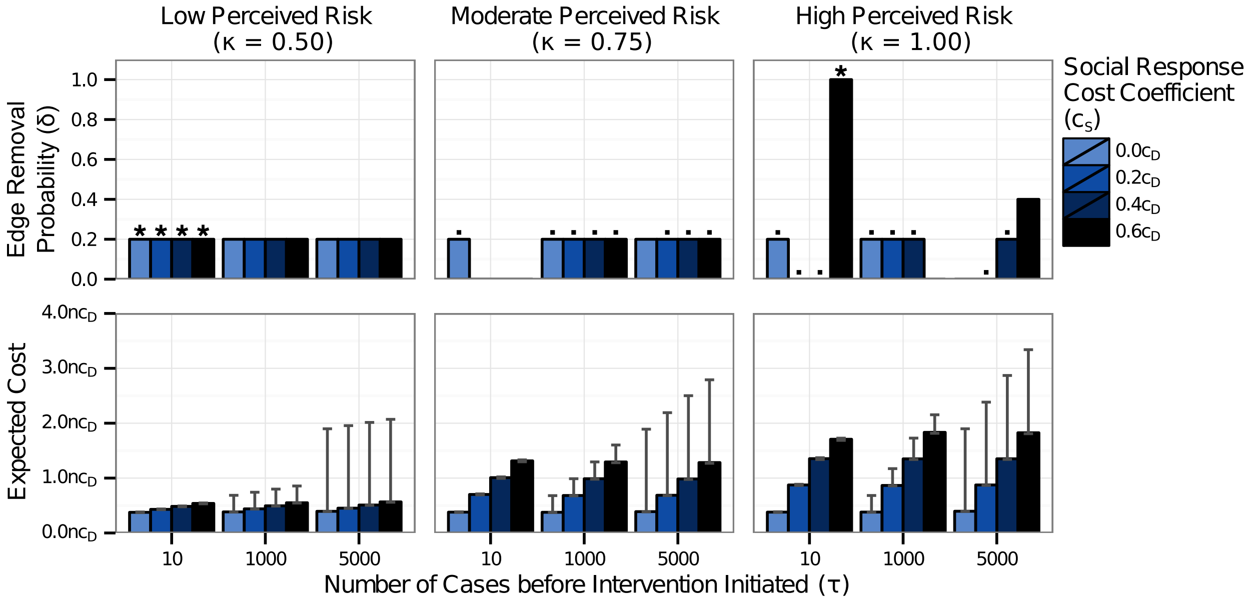

Figure S4. Most cost-effective intervention level and timing with no additional social response resulting from intervention for $T_I = 35$. The least cost level of edge removal and the associated expected cost are shown for three intervention thresholds ($\tau =$10 cases, 1000 cases, 5000 cases) and for four values of the cost coefficient of social response (c_S). The most cost-effective intervention is marked with a star (*) for each value of c_S. In cases where the pairwise difference in expected cost between strategies was not statistically significant with a permutation test, all least-cost strategies are marked with dots ($\cdot$). The error bars on the expected cost indicate the bootstrapped empirical 95% confidence interval.

References

1. Cauchemez S, Valleron A-J, Boëlle P-Y, Flahault A, Ferguson, NM. Estimating the impact of school closure on influenza transmission from Sentinel data. Nature. 2008; 452: 750-754. doi: 10.1038/nature/06732.

2. WHO Writing Group. Nonpharmaceutical interventions for pandemic influenza, national and community measures. Emerg Infect Dis. 2006; 12: 88-94. doi: 10.3201/eid1201.051371.
